# Supplementary material for: Meta-Analysis and Gene Set Enrichment Relative to ER Status Reveal Elevated Activity of MYC and E2F in the “Basal” Breast Cancer Subgroup
Source: PLoS One. 2009 Mar 9;4(3):e4710. doi: 10.1371/journal.pone.0004710 (PMC2650420; doi:10.1371/journal.pone.0004710)
Supplement: Dataset S1 — GSEA results from first screen. The file contains a zipped archive of the HTML results of the second GSEA screen. Reports for enriched or depleted datasets may be accessed from the “index.html” link within the my_analysis.GseaPreranked.1219975950625.rpt.zip ZIP archive. Enrichment plots are available for each of the 50 most enriched and depleted gene sets. (13.32 MB ZIP) [file pone.0004710.s001.zip › my_analysis.GseaPreranked.1219975950625.rpt.zip!//GREENBAUM_E2A_UP.html]

Details for gene set GREENBAUM\_E2A\_UP[GSEA]

|  || Dataset | zSco.ERneg.to.ERpos\_collapsed |
| Phenotype | NoPhenotypeAvailable |
| Upregulated in class | na\_pos |
| GeneSet | GREENBAUM\_E2A\_UP |
| Enrichment Score (ES) | 0.6508307 |
| Normalized Enrichment Score (NES) | 2.1589658 |
| Nominal p-value | 0.0 |
| FDR q-value | 0.0 |
| FWER p-Value | 0.0 |
Table: GSEA Results Summary

  

Fig 1: Enrichment plot: GREENBAUM\_E2A\_UP      
 Profile of the Running ES Score & Positions of GeneSet Members on the Rank Ordered List

  

| PROBE | GENE SYMBOL | GENE\_TITLE | RANK IN GENE LIST | RANK METRIC SCORE | RUNNING ES | CORE ENRICHMENT || 1 | TTK |  |  | 11 | 7.059 | 0.0591 | Yes |
| 2 | BUB1 |  |  | 41 | 6.445 | 0.1116 | Yes |
| 3 | CCNA2 |  |  | 52 | 6.297 | 0.1642 | Yes |
| 4 | EIF4EBP1 |  |  | 65 | 6.108 | 0.2152 | Yes |
| 5 | MKI67 |  |  | 98 | 5.871 | 0.2626 | Yes |
| 6 | KIF2C |  |  | 124 | 5.703 | 0.3091 | Yes |
| 7 | SLC7A5 |  |  | 235 | 5.169 | 0.3447 | Yes |
| 8 | KIF4A |  |  | 266 | 5.031 | 0.3851 | Yes |
| 9 | CCNB2 |  |  | 431 | 4.589 | 0.4117 | Yes |
| 10 | AURKA |  |  | 460 | 4.483 | 0.4476 | Yes |
| 11 | MAD2L1 |  |  | 477 | 4.446 | 0.4842 | Yes |
| 12 | PLK4 |  |  | 524 | 4.292 | 0.5171 | Yes |
| 13 | KIF11 |  |  | 713 | 3.909 | 0.5361 | Yes |
| 14 | TYMS |  |  | 829 | 3.738 | 0.5592 | Yes |
| 15 | NUSAP1 |  |  | 836 | 3.732 | 0.5904 | Yes |
| 16 | H2AFX |  |  | 930 | 3.591 | 0.6139 | Yes |
| 17 | PFKP |  |  | 1165 | 3.281 | 0.6241 | Yes |
| 18 | HS3ST1 |  |  | 1563 | 2.891 | 0.6188 | Yes |
| 19 | CCNB1 |  |  | 1626 | 2.823 | 0.6381 | Yes |
| 20 | EPRS |  |  | 2004 | 2.527 | 0.6311 | Yes |
| 21 | ECT2 |  |  | 2026 | 2.509 | 0.6508 | Yes |
| 22 | WBP5 |  |  | 2542 | 2.179 | 0.6306 | No |
| 23 | FGFR2 |  |  | 3146 | 1.868 | 0.6010 | No |
| 24 | CDKN2C |  |  | 3148 | 1.866 | 0.6168 | No |
| 25 | TOP2A |  |  | 3735 | 1.610 | 0.5864 | No |
| 26 | RACGAP1 |  |  | 4097 | 1.475 | 0.5717 | No |
| 27 | PTGS1 |  |  | 4669 | 1.264 | 0.5395 | No |
| 28 | PGK1 |  |  | 5836 | 0.865 | 0.4591 | No |
| 29 | SLC12A2 |  |  | 10723 | -1.332 | 0.1027 | No |
| 30 | SCIN |  |  | 11066 | -1.628 | 0.0908 | No |
| 31 | CA2 |  |  | 11917 | -2.453 | 0.0476 | No |
| 32 | KLHDC2 |  |  | 13305 | -6.820 | 0.0011 | No |
Table: GSEA details [plain text format]

  

Fig 2: GREENBAUM\_E2A\_UP: Random ES distribution      
 Gene set null distribution of ES for **GREENBAUM\_E2A\_UP**
